# A developmental formative evaluation of a pilot participatory music program for veterans with housing insecurity

**DOI:** 10.1186/s12889-023-16427-8

**Published:** 2023-08-19

**Authors:** Sally Wasmuth, Nicholas A. Rattray, Phillip Cheng, Shannon Crow, Jennifer Myers, Debra S. Burns, Laura J. Myers, Brittany Hook, Anne Lustig, Anthony J. Perkins, Ariel J. Cheatham, Dawn M. Bravata

**Affiliations:** 1 Department of Occupational Therapy, Indiana University-Purdue University, Indianapolis, IN USA; 2 Department of Veteran Affairs, Health Services Research and Development (HSR&D) Precision Monitoring to Transform Care (PRISM) Quality Enhancement Research Initiative (QUERI), United States, Richard L. Roudebush VA Medical Center, HSR&D Mail Code 11H, 1481 West 10th Street, IN 46202 Indianapolis, USA; 3 HSR&D Center for Health Information and Communication (CHIC), United States Department of Veteran Affairs, Richard L. Roudebush VA Medical Center, Indianapolis, IN USA; 4 Department of Internal Medicine, Indiana University School of Medicine, Indianapolis, IN USA; 5 Department of Anthropology, Indiana University-Purdue University, Indianapolis, IN USA; 6 School of Medicine, Indiana University, IN Indianapolis, USA; 7 Department of Music and Arts Technology, Indiana University-Purdue University, Indianapolis, IN USA; 8 United States Department of Veteran Affairs, Domiciliary Care for Homeless Veterans Program (DCHV), IN Indianapolis, USA; 9 Department of Biostatistics, Indiana University School of Medicine, Indianapolis, IN USA; 10 Department of Neurology, Indiana University School of Medicine, Indianapolis, IN USA; 11 Regenstrief Institute, Indianapolis, IN USA

**Keywords:** Housing insecurity, Homelessness, Music education, Formative evaluation

## Abstract

**Background:**

Interventions are needed to improve well-being and promote community reintegration among Veterans with housing insecurity. The objective was to conduct a developmental formative evaluation of a participatory music program.

**Methods:**

This single-site, pilot study implemented a participatory music program at a U.S. Department of Veterans Affairs (VA) Homeless Domiciliary that included one-hour sessions (group music instruction and ensemble playing), 3 times per week for 3 months. Intervention development was guided by the Model of Human Occupation (MOHO). Evaluation was guided by the MOHO and the Consolidated Framework for Implementation Evaluation (CFIR). Qualitative data were collected via semi-structured interviews from participants and non-participants, and were analyzed using an interdisciplinary, constant comparison qualitative analysis technique.

**Results:**

Sixteen program participants and 8 non-participants were enrolled, age range 26–59 (mean 41; standard deviation, 11) years; 75% were White. The sample for this study (*N* = 12) included five participants and seven non-participants. Semi-structured interview responses produced three salient themes illuminating Veterans’ perspectives: (1) key characteristics of the intervention (the relative advantage of the participatory program over other problem-focused programs; the importance of a supportive, encouraging teaching; the group setting; the role of music); (2) the therapeutic power of the program (based on it being enjoyable; and serving as an escape from preoccupations); and (3) the context and culture (which included Veterans supporting each other and the Domiciliary setting).

**Conclusions:**

Veterans described the benefits of a participatory music intervention compared to problem-based groups, which included enjoyment, skill acquisition facilitating pride, escape, reconnecting with their identity prior to current problems, and experiencing positive aspects of Veteran culture such as mutual support and discipline. These data support ongoing research about participatory music programs to support Veterans with housing insecurity.

**Graphical Abstract:**

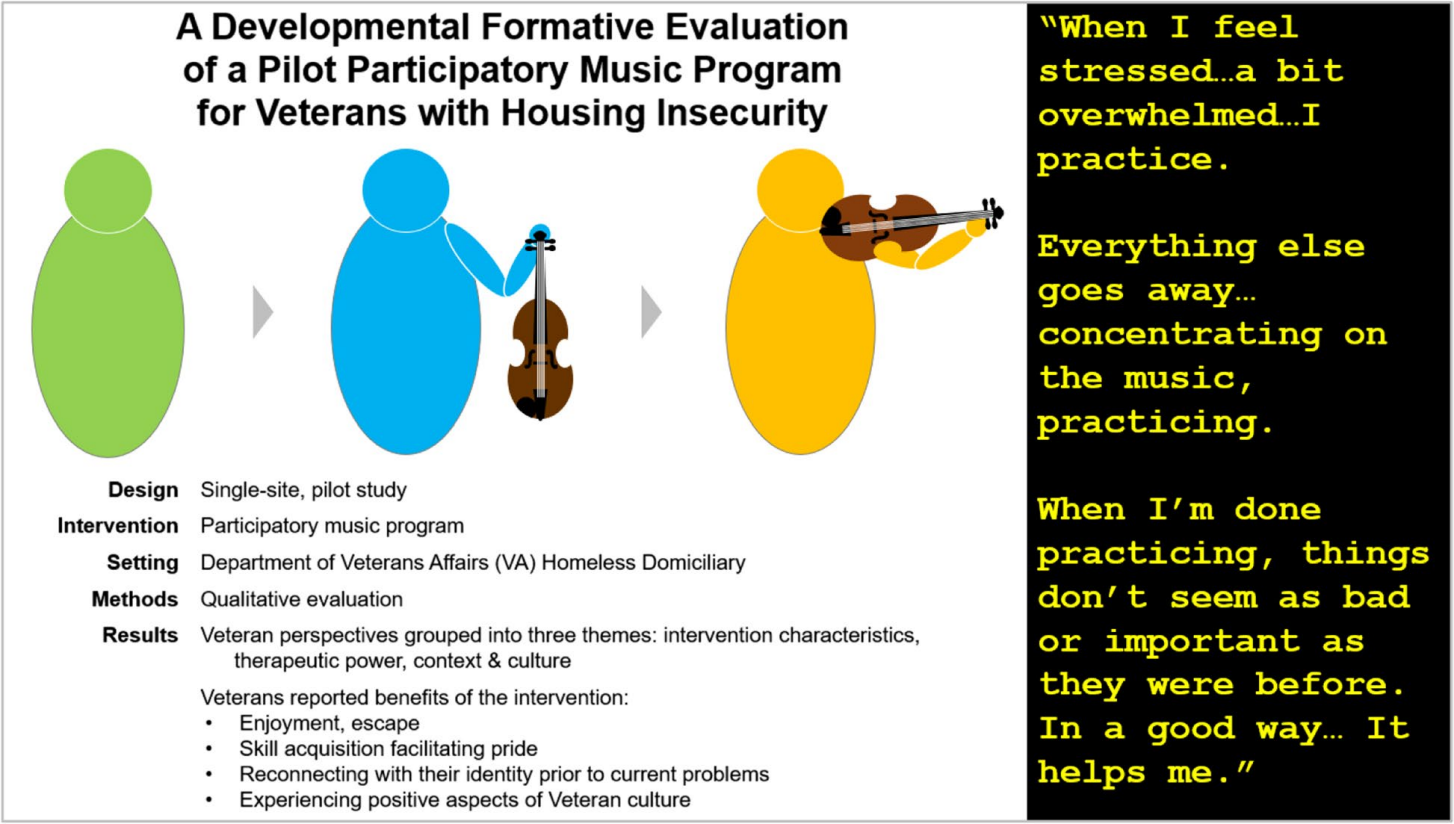

**Supplementary Information:**

The online version contains supplementary material available at 10.1186/s12889-023-16427-8.

## Introduction

On any given day, nearly 50,000 Veterans are homeless in the United States [1]. The Department of Veterans Affairs (VA) Domiciliary Care for Homeless Veterans Program provides residential care for Veterans with housing insecurity [2]. However, even with the support and resources provided at VA Domiciliaries, many Veterans with housing insecurity experience social isolation [3] and struggle to transition from the Domiciliary into the community, [4] illustrating a need for interventions to facilitate a successful transition and community reintegration [5].

Programs for persons with housing insecurity often include sports-based or arts-based interventions which serve as pleasurable leisure activities and which may enhance participation and community engagement [6, 7]. Participatory music programs hold promise in addressing social isolation and the related poor quality of life and difficulties with community reintegration for persons with housing insecurity [3, 8]. However, little research has examined the impact of a participatory music program amongst Veterans with housing insecurity.

This manuscript describes a developmental formative evaluation of a participatory music intervention to plan a future larger-scale study that would examine the potential effectiveness and implementation of a music-based intervention on community reintegration for Veterans with housing insecurity [9]. Our long-term goal was to develop a program that could potentially scale to both other VA Domiciliaries and to non-VA supportive housing settings. The objective of this developmental formative evaluation was to obtain the perspective of Veterans with housing insecurity to inform the design of the participatory music intervention.

## Methods

### Design: developmental formative evaluation

This single-site, non-randomized pilot project was conducted as a developmental formative evaluation with the goal of developing and iteratively refining the intervention program for a future, larger effectiveness and implementation study [9]. Developmental formative evaluations are conducted early in the research process and focus on enhancing the success of an intervention within a specific setting or context by examining potential barriers and facilitators to adoption of an innovation [9]. Developmental formative evaluations seek to identify and understand potential problems and possibly overcome them prior to initiating larger-scale implementation or research projects [9].

We sought the perspective of participants about barriers and facilitators of program engagement, their experience with the intervention sessions, and suggestions for program improvement. We also sought the perspectives of non-participants to improve our approach to recruitment and intervention design (e.g., suggestions for what might have made the program more appealing such that they would have considered participating). This pilot study was not designed to identify potential effects of the intervention on outcomes (e.g., community reintegration).

### Theoretical conceptualization

The participatory music education program was designed to provide a meaningful activity which had the potential to influence a person’s motivation, identity, and well-being [10]. The intervention was designed to give Veterans residing at the Domiciliary an opportunity to participate in an activity that might replace prior unhelpful habits (e.g., consuming alcohol when not otherwise engaged in a meaningful endeavor). Because the intervention sought to facilitate participation in a meaningful activity, the program aligned with a rehabilitation medicine framework, specifically the occupational therapy Model of Human Occupation (MOHO; Table 1) [11–13]. The MOHO is a widely applied, occupation-focused conceptual framework, that describes how participation in meaningful activities within specific environments leads to personal agency and adaptation, and also describes how personal attributes contribute to a person’s skill, performance, and participation [10].

**Table 1:** Mapping the intervention and the conceptual framework

Contributors to Occupational Performance	Intervention
**Volition** A person’s values, personal causation and interests [14]	The intervention seeks to influence participants’ interest and motivation by giving them a musical instrument as well as instruction in its use. The playing of an instrument may be viewed as a desirable skill, enhancing participants’ self-esteem.
**Habituation** The acquired and exhibited patterns of occupational performance based on habits and roles [14]	Offering the intervention sessions three times per week may facilitate forming a regular pattern of participation. Because participants keep their instruments for the duration of their involvement in the program, they may develop practice habits during their free time. Participants may see themselves in new roles, such as: learner, musician, or orchestra member.
**Performance Capacity** Ability to perform an activity based on one’s mental and physical capacities and lived experiences [14]	Providing music education may enhance participants’ skill and confidence in playing a stringed instrument.
**Environment** The physical and social aspects that impact how meaningful activities are motivated, organized, and performed [14]	The intervention has been designed to use both a group setting and a supportive teacher-student interaction which may promote social supports and motivation for engagement.

### Setting

The Richard L. Roudebush VA Homeless Domiciliary was a 50-bed residence for Veterans with housing insecurity. Veterans are adults who served in any of the branches of the US military (i.e., Army, Navy, Marines, Air Force, Non-Defense, Reserve Forces). Veterans include individuals who served in wartime (e.g., World War II, Korean Conflict, Vietnam Era, etc.) and during peacetime (e.g., pre-World War II, between the Vietnam Era and the Gulf War Era, etc.). As of September 2020, there were 19.4 million living US Veterans [15].

Veterans residing at the Domiciliary received interdisciplinary care that included medical, psychiatric, vocational, educational, recreational, and social services to address mental illness and substance abuse, co-occurring medical problems, and psychosocial needs. The Domiciliary interdisciplinary team did not include an occupational therapist but did include recreational therapists who offered both creative arts opportunities (e.g., shoe- and mask-making expressive arts activities) and recreational activities (e.g., bowling). Although the US active military and Veteran communities have a long-standing history of using music for therapeutic and recreational purposes, [16] the Domiciliary did not have any music-making programming. The intervention was implemented from 2018 to 2019 as an elective recreational rehabilitation opportunity. This study was prospectively registered with clinicaltrials.gov (NCT03653130) on 31/08/2018.

### Participant and non-participant eligibility

Program participants and non-participants were recruited from residents of the Domiciliary who were eligible for elective activities. Veterans were eligible for elective activities if they had been a resident for at least two weeks and had not committed any infractions. Generally, Veterans resided at the Domiciliary for six to nine months. Veterans with visual impairment, profound hearing impairment, cognitive impairment, or other conditions that might interfere with their ability to play an instrument were excluded.

### Participant and non-participant recruitment

Project staff attended the community meetings at the Domiciliary to inform the Veterans about the program, invite them to attend intervention sessions, and reiterate that participation was entirely voluntary; the music educator played short songs on the various instruments during these sessions. Posters about the program were placed in high-traffic areas within the Domiciliary. Participants were invited for interviews at the time of the music-making intervention sessions. Veterans who decided not to participate were invited for interviews at the community meetings. The latter were recruited from Veterans residing at the Domiciliary who were willing to be study participants but who chose not to participate in the music group.

### Intervention

The intervention was a participatory music program where participants engaged in music-making as members of a string orchestra. Participants received an instrument of their choice (violin, viola, or cello). One-hour lessons with a music educator experienced in adult music education occurred three times per week. Sessions included thirty minutes of group music instruction by followed by thirty minutes of ensemble participation [17, 18].

The repertoire was chosen by the music educator in consultation with participants. Participants were given sheet music for use during their own practice and group sessions. The music educator created instructional videos demonstrating how to play the instruments and providing practice suggestions. Participants were encouraged to practice during their free time.

The participatory music program focused on the use of stringed instruments for several reasons. Stringed instruments are appropriate in both solo and group settings. Many Domiciliary programs used a group format, which was familiar and comfortable to potential participants. Playing a stringed instrument within an orchestra setting may promote a sense of accountability, provide opportunities for social participation, and foster the development of a new role (“I am the orchestra’s cellist”). Stringed instruments are melodic (unlike a drum or a triangle which generally produces a single note), allowing participants to play music that is familiar, facilitating both enjoyment and skill acquisition. In addition, stringed instruments (in contrast to keyboards) facilitate learning and playing because the music is written in one line in a single clef. Finally, although cellos are larger, stringed instruments can be conveniently transported, even if using public transportation.

### Pilot project implementation

The music educator developed and facilitated the lessons because, based on the MOHO conceptualization, a key objective of the program was to provide participants with new skills. An occupational therapist and music therapist served as consultants on the implementation program team.

### Evaluation

The evaluation was guided principally by the MOHO framework, [11–13] supplemented with select constructs from the Consolidated Framework for Implementation Research (CFIR) [19]. Specifically, we examined the CFIR constructs related to intervention and organizational context.

#### Participants

We evaluated participants’ experiences of the program by collecting qualitative data from semi-structured interviews conducted one month after enrollment. Veterans who participated in the program were asked about past music education, barriers and facilitators of program participation, experience in intervention sessions, perceptions of their membership in the orchestra, and suggestions for program improvement (see Supplement A for interview guide).

#### Non-participants

Veterans who did not participate in the program were invited for a one-time semi-structured interview which asked about barriers to participation, suggestions for whether or how the program could have been modified to overcome those barriers, and experiences with music (see Supplement B for interview guide).

#### Qualitative analysis

The qualitative researchers were from a diverse range of fields including occupational therapy (SW, female, interested in occupational therapy approaches to promote social justice), anthropology (NAR, male, interested in Veteran community reintegration), social work (JM, female, interested in implementation of innovative interventions for Veterans with unmet social needs), music education (SC, female, interested in the application of music-based interventions to improve well-being), and medicine (DMB, female, interested in developing and evaluating interventions to improve patient outcomes). The interviews were conducted by NAR, JM, AJC, and DMB; none of whom had prior relationships with the interviewees but all of whom had experience with qualitative methods. During the interviews only the Veteran and the interviewer were present. Participant interviews took approximately 30 min, non-participant interviews took approximately 15–20 min.

The interviews were audio-recorded, transcribed, and reviewed by study team members (all of whom had previous experience with qualitative methods) to ensure accuracy. Transcripts were imported into NVivo11 for analysis using the constant comparison technique [20]. This method involves: inductive coding, refinement of categories, exploration of relationships across categories, and integration of data. They independently analyzed several transcripts and then met to compare coded data and develop a codebook. The team drew on coded excerpts and analytic memos to form consensus on relevant themes while recursively engaging in epistemological dialogue to accommodate a plurality of perspectives regarding emerging concepts. With an established codebook, the team then independently coded the remainder of the transcripts, after which they met for cross-checking—examining whether different researchers coded the same data in the same way and discussing any discrepancies. Trustworthiness was supported by investigator triangulation where distinct and diverse perspectives are applied to qualitative data [21]. The application of investigator triangulation was relevant to this study where an occupational therapy framework was used to develop a music-making intervention. Our multi-disciplinary team’s engagement in investigator triangulation allowed us to examine emerging themes from multiple perspectives to develop a robust and comprehensive understanding of each construct. The study received Institutional Review Board (IRB) approval.

## Results

A total of *N* = 16 participants enrolled in the program. Participants ages ranged from 26 to 59 years: mean age 41 (standard deviation, 11) years. The majority of participants were White (12/16; 75%). Comorbidity was common among participants: 94% had substance use disorder, 69% had depression, 56% had anxiety, 44% had post-traumatic stress disorder (PTSD), 44% had a history of suicidality, and 44% had chronic pain (Table 2). The sample for the qualitative analysis that formed the basis for the formative evaluation comprised a total of *N* = 12 Veterans who agreed to be interviewed: *N* = 5 participants and *N* = 7 non-participants.

**Table 2:** Baseline characteristics

Characteristic	Participants (*N* = 16)	Non-Participants (*N* = 8)
Age (years): range	26–59	45–61
Mean ± standard deviation	41 ± 11	55 ± 5
Race: N (%)		
White	12 (75.0)	3 (37.5)
Black	1 (6.3)	4 (50.0)
Other/unknown	3 (18.8)	1 (12.5)
Male sex: N (%)	16 (100)	7 (87.5)
*Comorbidity*: N (%)		
Depression	11 (68.8)	3 (37.5)
Anxiety or panic	9 (56.3)	2 (25.0)
Post-traumatic stress disorder (PTSD)	7 (43.8)	2 (25.0)
Suicidality: ideation or attempt	7 (43.8)	1 (12.5)
Substance use disorder	15 (93.8)	6 (75.0)
Chronic pain	7 (43.8)	5 (62.5)
Tobacco use	9 (56.3)	2 (25.0)
Duration of domiciliary stay (months): range	2.8–12.6	1.5–8.4
Mean ± standard deviation	6.4 ± 2.8	6.0 ± 2.4

Interview responses illuminated Veterans’ perspectives and experiences of the participatory music program. Responses produced three salient overarching themes: (1) intervention, (2) therapeutic power, and (3) context. These themes are described and expanded below with exemplary quotes organized under subthemes (Table 3).

**Table 3:** Veterans perspectives and experiences of the participatory music program

Theme	Domain	Illustrative Quotations	MOHO^a^ Construct
**Intervention**	Relative Advantage	“I thought it was a unique program. Different from a lot of the other programs that the VA offers; that will really help the Veterans.” “Other classes are just kind of the same stuff put in different ways; so it’s kind of redundant at times.”	**Volition**: positive perspectives about the program as a key motivator for participation
	Teacher	“[The teacher] was a big part of why I went down there in the first place, because she has that personality that just, everybody wanted, even the other guys, they want to go down and just see what it’s all about because of her passion.”	**Volition**: positive impressions about the teacher as a key motivator for participation
	Group	“It’s always fun to play with a big group…It’s enjoyable, especially to be around other Veterans. It’s kind of familiar, you know, to have that camaraderie again.”	**Environment**: social context of the program
		“[I would] prefer the 1-on-1, so it allows me to go at a quicker pace. I don’t have to wait on people to catch up.”	**Performance Capacity**: personal preference and attitude facilitate skill acquisition and performance
	Music	“Music is life. It’s living. It’s an expression of character, of mood.” “I’m not a musical; I don’t like music.”	**Volition**: personal characteristic (perspectives about music) influence participation
**Therapeutic Power**	Enjoyment	“I got joy out of it.” “It’s enjoyable…it’s fun.” “I have loved participating! I hope it continues for a long time.”	**Volition**: the fun of the experience motivated participation in the program
	Escape from Preoccupations	“When I feel stressed…a bit overwhelmed…I practice. Everything else goes away… concentrating on the music, practicing. When I’m done practicing, things don’t seem as bad or important as they were before. In a good way… It helps me.”	**Habituation**: the practice (habit) leads to personal agency and adaptation
**Context & Culture**		“…other guys even though they didn’t participate, they were pretty supportive. I think that’s just the Veteran mentality when they see somebody trying to do something, whether they do it or not, they will support.” “Orchestra is for suburban white people”	**Environment**: examples of social context (Veteran culture, race, socioeconomic status)

^a^MOHO refers to the Model of Human Occupation which was the conceptual framework grounding the project

### 1. Intervention

Data within the theme ‘intervention’ detailed Veterans’ perceptions of the program. Participants described several aspects of the participatory music program such as the activity of music-making and the qualities of the teacher that were unique and useful and/or that gave it relative advantage over other types of programming.

#### Relative advantage

Some Veterans compared the participatory music program to other services available to them, suggesting it had relative advantage for several reasons such as its uniqueness and its emphasis on learning to play music, rather than discussing personal problems, for example. “*I thought it was a unique program. Different from a lot of the other programs that the VA offers; that will really help the Veterans.*” Another noted, “*Other classes are just kind of the same stuff put in different ways; so it’s kind of redundant at times*,” and, “*It’s just kind of a break from all that where you’re just learning to play music. You’re not, you know, you’re not talking about your feelings and all that kind of hippie stuff.*” That participant specifically described learning to play music as “*beneficial to anybody.*”

#### Teacher

Several Veterans noted that the teacher had qualities which significantly influenced their experience of the intervention, noting she *“…was a big part of why I went down there in the first place, because she has that personality that just, everybody wanted, even the other guys, they want to go down and just see what it’s all about because of her passion*.” Another expressed how he “…*never felt like she was judging me or critiquing me*” and another explains *“[name]’s a great teacher. She’s very enthusiastic. She’s also really funny. She’s very helpful.*”

#### Group

When describing the intervention, Veterans also commented on both the positive and negative aspects of it being a group intervention. For instance, one expressed “*It’s always fun to play with a big group…It’s enjoyable, especially to be around other Veterans. It’s kind of familiar, you know, to have that camaraderie again.*” By contrast, another was frustrated with the group, explaining he would “*prefer the 1-on-1, so it allows me to go at a quicker pace. I don’t have to wait on people to catch up*.”

#### Music

Veterans also suggest a person’s appreciation of music (or lack thereof) influenced the likelihood of participating in and/or enjoying this intervention. For example, one participant noted “*Music is life. It’s living. It’s an expression of character, of mood*” and in contrast, another said “*I’m not a musical; I don’t like music*.” Interviewees also made suggestions about specific instrument preferences. For instance, one noted: “*Add some guitar*” and another explained: “*I didn’t think that I could be very good with the violin. So I did the viola, and I didn’t want the cello because it was sort of big.”* One participant described enjoying the personal agency allowed during the process of choosing an instrument: “*We were allowed to pick whichever one, so it was kind of pick it up, play around with it and get a feel for it and decide from there*.”

### 2. Therapeutic power

The second major theme – therapeutic power – included data describing how participation in the intervention impacted participants’ lived experiences. Veterans described two major ways that the intervention fostered well-being: by providing opportunities for enjoyment and escape.

#### Enjoyment

As noted above, Veterans found this intervention to be helpful in that it provided a source of enjoyment, which contrasted with other opportunities at the Domiciliary that were more problem-based. Participants noted: “*I got joy out of it,” “It’s enjoyable…it’s fun,” and “I have loved participating! I hope it continues for a long time.”* Enjoyment was described in relation to reclaiming identity and connecting with others – the enjoyment experienced in the group was also motivating: “*It reminded me of my past a little bit. Going to New York and seeing shows on Broadway and listening to the orchestra music. I had forgotten about those good times because of my situation. It reminded me that I need to get back to a place where I can enjoy going to shows and listening…It helped my self-esteem…I got in touch with who I used to be before I had all these issues.”* One Veteran, in response to the probe “What were your most favorite parts of the group?” expressed joy in accomplishing the skill of “*Learning to read music!”*

#### Escape from preoccupations

In addition to enjoyment, participants noted that the intervention provided some relief from stress or negative emotions. “*When I feel stressed…a bit overwhelmed…I practice. Everything else goes away… concentrating on the music, practicing. When I’m done practicing, things don’t seem as bad or important as they were before. In a good way… It helps me.*” It provided a way for participants to distance themselves from pressing problems. “*It just allows you to escape whatever you’re going through during the day.*” Another noted, “*I think that it’s relaxing. It helps with stress and anxiety*.” Further illustrating this, another explained, it “*Let’s you just kinda get out of yourself for a while and just focus on music*.” These data suggested the intervention could provide relief from problems, even if it did not directly aim to remediate specific problems. The intervention offered an alternative to problem-based approaches by engaging participants in something meaningful, relaxing, and enjoyable that distracted Veterans from their problems for a time.

### 3. Context & culture

The third main theme categorized data describing cultural and contextual factors that further influenced how Veterans experienced or perceived the intervention. For example, Veterans listed several cultural factors influencing how the program was perceived, providing suggestions to make it more appealing: one noted “*Orchestra is for suburban white people*” and “‘*Orchestra’ is a bad name…’Music Program’ is better*.” Several participants expressed positive sentiments toward playing “*the fiddle*” and chose the violin because of it.

Also, regarding culture, participants noted both Veteran culture and the culture of the domiciliary as facilitating and/or supporting participation in the program. For example, one described how at the domiciliary *“…other guys even though they didn’t participate, they were pretty supportive. I think that’s just the Veteran mentality when they see somebody trying to do something, whether they do it or not, they will support*.” Expanding on Veteran culture, another noted: “*most of us are Veterans, and well, and we already have a lot of discipline, but it kind of shows you that you are able to do anything if you really set your mind to it*.”

Regarding context, Veterans noted both personal and programmatic contextual factors. The personal context subtheme included concepts related to Veterans’ medical or mental health status (e.g., anxiety), fears, homeless status, and/or status in the domiciliary program. Some expressed having “*A lot of fear*” or being concerned that “*I probably won’t have no patience for it.*” Regarding programmatic context, participants noted that the fact that “*it counts as a class*” in terms of domiciliary programming was motivating and helpful. However, they also noted several competing programs – “*Just a lot of other activities going on*” – and difficulty with participating as they moved to higher levels within the Domiciliary when they were expected to be out working or job seeking.

## Discussion

Our pilot data suggest that the participation-based nature of this music program contributed to wellness. A key finding was that Veterans described the benefits of the program that was participatory rather than problem-based, offering opportunities for enjoyment and escape. Veterans distinguished the music group from other programs that asked them to reflect and focus on negative life situations. As part of the core therapeutic activities that residents of the Domiciliary were required to attend, Veterans were asked to identify key decisions, life-events, and behaviors that led them to their current situation; this process often required the Veterans to acknowledge that their actions negatively influenced relationships, employment, and housing. By contrast, the music group was fun allowed them to recall a time before they had their current problems. This reconnection with their former selves was described as a motivating push to get back to enjoying life and remembering who they were. Social participation and leisure activities have been reported to be more supportive of recovery than problem-based approaches [22]. These pilot findings suggest that Veterans with housing insecurity might benefit from participation-based programming rooted in the therapeutic power of engaging in meaningful occupations [23] to complement the existing problem-based activities.

Achieving new skills such as the ability to read music, alongside the supportive encouragement of other Veterans, highlighted existing, positive qualities of Veteran culture: discipline and mutual support [24]. Participating in executive challenges and socially engaging occupations can support community engagement and recovery [22, 25].

Veterans described several aspects of the intervention that may facilitate wellness, such as agency, personal choice, and the therapeutic power of music. The music program provided a coping strategy—it was a relief from negative preoccupations. In addition, the music instructor was perceived as non-judgmental, facilitating trust and participation despite Veterans’ anxiety or fear they would not succeed. Kopacz et al. [26] found that trust correlated with several positive health outcomes in Veterans with PTSD, indicating the importance of this connection the music program instructor established with participants.

Our findings highlighted several contextual factors to consider in a future clinical trial. Veterans noted the importance of language in describing the program (e.g., the ‘fiddle’ was more attractive than the ‘violin’; ‘music program’ was preferred to ‘orchestra’). These data underscored the need for the program design and delivery to be informed by diverse perspectives. The changing contexts of domiciliary residents illustrated how engaging in something beneficial such as the music program became more challenging as Veterans progressed in the domiciliary program and they were expected to hold jobs or be looking for work full-time. These observations are supported by other studies that have described the early recovery period as one of occupational deficit, but that as recovery progresses, occupations and their concomitant demands return, potentially making participation in wellness activities more challenging both in terms of time management and motivation [27].

The MOHO conceptual framework was useful in making sense of the Veterans’ diverse perspectives about the program and why they choice to participate or not participate. We provide three examples of how considering the MOHO domains influenced our understanding of the qualitative results. First, when designing the program, we had conceptualized the habituation domain of the MOHO in terms of providing encouragement and opportunities for practice playing the instruments. We anticipated that program engagement and independent practice would lead to skill acquisition (i.e., the ability to play a song) which would in turn promote positive self-image. In addition, one of the explicit goals of the therapists at the Domiciliary is to provide residents with opportunities to form new meaningful habits as part of their healing journey—which aligned with the MOHO construct of habituation. However, the results revealed that practice was helpful, not via enhanced musical proficiency as we had expected, but rather through relaxation, enjoyment, and distraction. Second, the MOHO volition domain makes explicit motivations for participation. By interviewing both participants and eligible persons who chose not to participate, we learned about program characteristics that either promoted or hindered participation—key knowledge as we seek to improve the program. Third, Veterans with housing insecurity who resided at the Domiciliary had both physical limitations and mental health related barriers to engaging in the music education program. Regarding the performance capacity MOHO domain, we had expected that Veterans with the greatest performance capacity would be the ones most likely to engage in and benefit from the program. However, the results indicated that patients with limitations that potentially inhibited performance capacity (e.g., anxiety) were the ones who reported genuine relief from participation. Practitioners and investigators are encouraged to consider the MOHO in future projects that seek to engage participants in a new activity.

### Limitations

Several limitations merit description. First, this pilot project was implemented within a VA Domiciliary and therefore may not be generalizable to other populations (e.g., non-Veterans, those without housing insecurity) or to other settings. Second, the music instructor was universally regarding positively by program participants, highlighting the importance of selecting instructors with similar appeal in future trials, and the need to carefully evaluate program fidelity and participants’ impressions of the instructor given the variability among potential interventionists and their approaches to music instruction. Third, these pilot data cannot be used to describe the potential role that a music-based program might play in promoting community reintegration because we did not have data from Veterans who transitioned out of the Domiciliary and into community housing. A future study is needed to obtain these key data. Fourth, the qualitative data were obtained from self-report. We sought to minimize possible recall bias in the self-reported data by conducting interviews either immediately after intervention sessions (for participants) or immediately following the community meetings (for non-participants). We sought to minimize potential social desirability bias in the self-reported data by using interviewers who were not part of the intervention program and by explicitly soliciting both positive and negative feedback. Finally, the participants of this study were mostly white men and thus findings reported in this study do not adequately capture the diversity of experiences and contexts within which the participatory music program may be perceived and experienced. Future trials will need to devote significant attention to the recruitment of Black, Indigenous, and other people of color as well as women and gender diverse individuals to fully represent the impact of this program on diverse populations of Veterans.

## Conclusion

Qualitative evaluation of a participatory music program to support Veterans with housing insecurity reflected several important elements to consider in a future clinical trial. Veterans enjoyed the program and valued the participation-focused rather than problem-focused groups. The universal positive regard for the music instructor illustrated the importance of ensuring that interventionists are supportive and non-judgmental. Future studies seeking to implement programs within the VA Domiciliary setting will have to consider how best to integrate the program in ways that complement rather than conflict with existing offerings. These data support ongoing research into the effectiveness of participatory music programs to support Veterans with housing insecurity.

### Supplementary Information

**Additional file 1.**

**Additional file 2.**
