# Supplementary material for: A developmental formative evaluation of a pilot participatory music program for veterans with housing insecurity
Source: BMC Public Health. 2023 Aug 19;23:1583. doi: 10.1186/s12889-023-16427-8 (PMC10439562; doi:10.1186/s12889-023-16427-8)
Supplement: Supplementary file 1 — Additional file 1. [file 12889_2023_16427_MOESM1_ESM.doc]

**Participatory Music Program for Homeless Veterans (Crossroads Orchestra):**

**Pilot Evaluation**

**INTERVENTION SEMI-STRUCTURED INTERVIEW GUIDE**

Thank you for agreeing to participate in an interview. Our team is interested in hearing more about your perspective on participating in the Crossroads group. We will ask questions about your background, your experiences in the program, what you enjoyed about the program, what was challenging about the program, and any suggestions you have for improving the program. Participation in this interview is voluntary. Please let me know if you are uncomfortable or if you do not want to answer a certain question.

**FOR VETERANS WHO PARTICIPATE IN THE INTERVENTION**

What were some of your reasons for agreeing to participate in the Crossroads Orchestra group? What was your first impression? What did you think of what you heard when we came to talk about the program at the Dom community meeting?

How would you describe your experiences in the group sessions so far?

Additional probes:

- Most favorite things about group sessions
- Least favorite things about group sessions
- Can you think of a specific example of something you or didn’t like?
- Music education component
- Playing the instrument in a group
- Being in a group with other Veterans
- Session location (Domiciliary vs Regenstrief/VAMC if appropriate)

We are interested in your impressions of the music educator. Please describe your experiences working/learning with Shannon

Additional probes:

- Style of teaching
- Pace of learning
- Would you have preferred more or fewer sessions?
- Should there be more or less homework?
- Enough individual attention?
- Like or dislike music choices? Suggestions?

Do you practice your instrument or do anything to care for your instrument during your free time? Can you describe what you do in terms of practice outside of the program.

Additional probes:

- Where does practice take place?
- What is practiced?
- How much time is spent practicing?
- Is practice alone or with others?

Are there any aspects of the program that have made it challenging to participate? Have there been any barriers related to your participation in the Crossroads Orchestra program? **(If yes)**

- Probes
  - Learning a new skill
  - Playing an instrument in front of peers

How did you deal with those barriers? Was there something that the program could have done/offered to help deal with those barriers?

Do you feel like participating in the group is changing the way you see yourself or the way other people see you?

Additional probes:

- To what extent do you see yourself as a musician?
- How does being a member of the group affect the way you feel about yourself?

How would you describe what music means to you? Is there a word that comes to mind when you think about music? Do you consider yourself a “musical” person?

Have you ever played an instrument before? Have you ever had any kind of music education, including when you were a child?

Additional probes:

- Childhood music education (either through school or outside of school)
- Playing an instrument in a school band or taking band class
- Formal or informal music education
- Instruments played
- Duration of experience playing an instrument
- How long ago was the last time they played?
- Read music?

Do you have fun participating? What are the most fun aspects? What do you like most?

Is there any aspect of participating that is really challenging for you? What is the most challenging?

Additional probes:

From Time Use Interview:

- If you were not participating in Crossroads, what else might you be doing with this time?
- What do you do with your spare time? What types of hobbies are you interested in?
  - Prompts: Movies, sports, restaurants, outdoor trips
- Do you participate in any community-based activities?
  - Religious services, sports, Veterans council, choir
- How much time do you spend socializing?
  - Prompts: In a typical week?
  - With friends? Family?
- Are you currently employed or attending school?
  - Prompts: Job title, certification or degree, part time or full time

If the Crossroads Orchestra program were routinely scheduled, and available in the community, would you want to keep participating? What factors or supports would make it easier for you to continue to participate?

Could you describe the time since you separated from military service? How long has it been since you separated?

- What are things that you are working on currently in terms of personal goals ?
- In considering your experience since you separated from military service, can you describe how you came to join the domiciliary? How has your time here fit into your overall experience in reintegration or transitioning to being a civilian?
- What has being part of this community meant to you? Are there other communities or groups that are meaningful to you?
- Has being part of Crossroads affected your willingness to try new things?

Do you have any other comments that you would like to share that we have not asked about?

Additional probes:

- Suggestions for improvement
- Things you really liked or disliked about the program
- Would you recommend this program to others? Who could benefit from it, and how would you describe to someone who was not familiar with it?
