# Supplementary material for: A developmental formative evaluation of a pilot participatory music program for veterans with housing insecurity
Source: BMC Public Health. 2023 Aug 19;23:1583. doi: 10.1186/s12889-023-16427-8 (PMC10439562; doi:10.1186/s12889-023-16427-8)
Supplement: Supplementary file 2 — Additional file 2. [file 12889_2023_16427_MOESM2_ESM.docx]

**Participatory Music Program for Homeless Veterans (Crossroads Orchestra):**

**Pilot Evaluation**

**CONTROL SEMI-STRUCTURED INTERVIEW GUIDE**

Thank you for agreeing to participate in an interview. Our team is interested in hearing more about your perspective on the Crossroads group. We will ask questions about your background, why you chose not to join the Crossroads program, some barriers you may face, and any suggestions you have for improving the program. Participation in this interview is voluntary. Please let me know if you are uncomfortable or if you do not want to answer a certain question.

**FOR VETERANS WHO DECLINED TO PARTICIPATE IN THE INTERVENTION**

What were some of your reasons for deciding not to participate in the Crossroads Orchestra program? What was your first impression? What did you think of what you heard when we came to talk about the program at the Dom community meeting?

What can you tell us about your impressions of the Crossroads group so far? Have you heard the group playing their instruments at the Dom?

How would you describe your experiences completing our surveys so far?

Additional probes:

- Are there any aspects that you enjoy?
- Is there anything that we could do better?

Were there any specific challenges or barriers that kept you from joining the Crossroads Orchestra program? Was there something that the program could have done/offered to help deal with those barriers?

Do you feel like completing our surveys is affecting the way you see yourself or the way other people see you?

Additional probes:

- Does participating in research affect the way you feel about yourself?

How would you describe what music means to you? Is there a word that comes to mind when you think about music? Do you consider yourself a “musical” person?

Have you ever played an instrument before? Have you ever had any kind of music education, including when you were a child?

Additional probes:

- Childhood music education
- Playing an instrument in a school band or taking band class
- Formal or informal music education
- Instruments played
- Duration of experience playing an instrument
- How long ago was the last time they played?
- Read music?

From Time Use Interview:

- What types of activities do you do in your spare time?
  - Prompts: Movies, sports, restaurants, outdoor trips
- Do you participate in any community-based activities?
  - Religious services, sports, Veterans council, choir
- How much time do you spend socializing?
  - Prompts: In a typical week?
  - With friends? Family?
- Are you currently employed or attending school?
  - Prompts: Job title, certification or degree, part time or full time

If the Crossroads Orchestra program were routinely scheduled, and available in the community, would you want to keep participating? What factors or supports would make it easier for you to continue to participate?

- What are some of personal goals currently?
- In considering your experience since you separated from military service, can you describe how you came to join the domiciliary? How has your time here fit into your overall experience in reintegration or transitioning to being a civilian?
- What has being part of the community meant to you?

Do you have any other comments that you would like to share that we have not asked about?

Additional probes:

- Suggestions for improvement
- Things you really liked or disliked about the program
